# Commentary: Targeting angiogenesis in gastrointestinal tumors: strategies from vascular disruption to vascular normalization and promotion strategies angiogenesis strategies in GI tumor therapy

**DOI:** 10.3389/fimmu.2026.1790915

**Published:** 2026-02-26

**Authors:** Yaoyao Zhang, Jiyuan Ding

**Affiliations:** Department of Oncology and Hematology,Hangzhou Red Cross Hospital, Hangzhou, Zhejiang, China

**Keywords:** angiogenesis, commentary, future directions, gastrointestinal tumors, therapeutic strategies

## Introduction

The recent review by Li et al. in Frontiers in Immunology, “Targeting angiogenesis in gastrointestinal tumors: strategies from vascular disruption to vascular normalization and promotion strategies angiogenesis strategies in GI tumor therapy,” offers a systematic overview of molecular mechanisms, current therapeutic approaches, and future prospects in angiogenesis-targeted therapy for GI cancers (1). The authors notably emphasize the dual strategy of combining vascular disruption with normalization. This perspective merits further clinical and translational attention. However, the article still has some limitations worth exploring further in terms of breadth of coverage, research depth, and clinical translation aspects. Therefore, this commentary aims to expand the discourse by identifying research gaps and proposing actionable directions that were not fully addressed in the original review. These limitations and proposed directions represent key areas for future research breakthroughs.

## Subsections relevant for the subject

First, the article exhibits gaps in disease models and population coverage. The study primarily relies on evidence from adult gastrointestinal tumors and fails to adequately incorporate pediatric patient populations, such as neuroblastoma and hepatoblastoma, as well as certain tumor subtypes characterized by unique angiogenic features, like HPV-associated squamous cell carcinoma of the head and neck. Tumors of different ages and molecular subtypes exhibit significant differences in angiogenesis-driven mechanisms, microenvironment composition, and therapeutic response patterns. Consequently, ignoring this heterogeneity may limit the generalizability of research conclusions and may impact the development of stratified treatment strategies (2).

Secondly, the study still has scope for further development regarding the depth and integrative nature of its mechanistic research. Although it focuses on classical pathways, such as VEGF, it provides limited coverage of non-classical mechanisms in tumor angiogenesis (e.g., vascular mimicry (3), endothelial-mesenchymal transition (4)) and multicellular interaction networks (e.g., signaling crosstalk between endothelial cells, immune cells, and stromal cells (5)). Furthermore, current analyses predominantly rely on transcriptomic data. Future efforts should emphasize the integration of spatial multi-omics technologies to systematically elucidate the regulatory panorama of vascular functional states at the protein, metabolic, and epigenetic levels; this approach will facilitate the identification of more precise intervention targets.

Furthermore, the clinical translation perspective of the article could be further strengthened. On one hand, although the combination therapy strategy was mentioned, its practical feasibility was inadequately explored. Issues such as the high cost of combination therapies, accessibility across different healthcare systems, and the lack of dynamic biomarkers to predict vascular normalization efficacy were not sufficiently addressed. On the other hand, the promising natural compounds (e.g., Britanin) face core bottlenecks of low bioavailability and formulation instability, while engineered solutions such as nanoparticle delivery systems and structural modifications were not sufficiently developed. Additionally, interdisciplinary frontiers, such as gut microbiota-regulated angiogenesis and biomaterial-mediated local vascular reconstruction, were mentioned but did not culminate in a systematic approach for clinical translation.

## Discussion

The review by Li et al. provides a solid foundation for understanding angiogenesis-targeted therapies in GI cancers. By further investigating the outlined areas—particularly pediatric tumor specificity, cost-effectiveness, biomarker development, multi-omics integration, microbiota modulation, resistance mechanisms independent of VEGF, and natural compound formulation—the field can move toward more precise, personalized, and accessible treatment paradigms. Interdisciplinary and translational studies will be essential to fully realize the potential of anti-angiogenic strategies to improve patient outcomes in GI cancers.

To provide a clearer perspective on future development directions, the conclusion section of the article could be more forward-looking and structured. It is recommended to propose a phased research roadmap. First, prioritize the use of single-cell and spatial transcriptomics technologies to map endothelial cell heterogeneity. Next, develop a dynamic monitoring system based on liquid biopsy and radiomics to enable early prediction and intervention of treatment responses. Finally, utilize biomimetic models such as organoids and organ-on-a-chip to conduct high-throughput screening of personalized combination therapies in simulated tumor microenvironments, thereby truly advancing vascular-targeted therapy to a new stage of precision and efficiency.

## References

[1] Li J Li Z Wang K . Targeting angiogenesis in gastrointestinal tumors: strategies from vascular disruption to vascular normalization and promotion strategies angiogenesis strategies in GI tumor therapy. Front Immunol. (2025) 16:1550752. doi: 10.3389/fimmu.2025.1550752, PMID: 40330478 PMC12052729

[2] Lugano R Ramachandran M Dimberg A . Tumor angiogenesis: causes, consequences, challenges and opportunities. Cell Mol Life Sci. (2020) 77:1745–70. doi: 10.1007/s00018-019-03351-7, PMID: 31690961 PMC7190605

[3] Bai J Xu Z Liao C Yang L . Research progress in vasculogenic mimicry in multiple tumors. Zhong Nan Da Xue Xue Bao Yi Xue Ban. (2017) 42:357–64. doi: 10.11817/j.issn.1672-7347.2017.03.020, PMID: 28364113

[4] Watabe T Takahashi K Pietras K Yoshimatsu Y . Roles of TGF-β signals in tumor microenvironment via regulation of the formation and plasticity of vascular system. Semin Cancer Biol. (2023) 92:130–8. doi: 10.1016/j.semcancer.2023.04.007, PMID: 37068553

[5] Xu J Ding L Mei J Hu Y Kong X Dai S . Dual roles and therapeutic targeting of tumor-associated macrophages in tumor microenvironments. Signal Transduct Target Ther. (2025) 10:268. doi: 10.1038/s41392-025-02325-5, PMID: 40850976 PMC12375796

